# Patient Involvement in Decisions regarding Emergency Department Discharge: A Multimethod Study

**DOI:** 10.1155/2023/4997401

**Published:** 2023-06-06

**Authors:** Marie Louise Thise Rasmussen, Kirsten Lomborg, Kasper Karmark Iversen, Hanne Konradsen

**Affiliations:** ^1^Department of Emergency Medicine, Copenhagen University Hospital-Herlev and Gentofte, Herlev 2730, Denmark; ^2^Department of Clinical Research, Copenhagen University Hospital-Steno Diabetes Center Copenhagen, Herlev 2730, Denmark; ^3^Department of Clinical Medicine, Faculty of Health and Medical Sciences, University of Copenhagen, 2200 Copenhagen N, Denmark; ^4^Division of Nursing, Department of Neurobiology, Care Sciences, and Society, Karolinska Institute, Stockholm 171 77, Sweden; ^5^Department of Gastroenterology, Copenhagen University Hospital-Herlev and Gentofte, Herlev 2750, Denmark

## Abstract

**Background:**

Unmet care needs and more than one reasonable discharge solution have been identified among patients in the emergency department. Less than half of the patients attending emergency care have reported being involved in decisions to the degree they have wanted. Having a person-centered approach, such as involving patients in decisions regarding their discharge, has been reported as being associated with beneficial outcomes for the patient.

**Aim:**

The aim of the study was to explore the extent of patients' involvement in discharge planning in acute care and how patient involvement in decisions regarding discharge planning is managed in clinical practice.

**Methods:**

A multimethod study, including both quantitative and qualitative data, was carried out. The quantitative part included a descriptive and comparative analysis of additional data from the patient's medical records and patient's responses to the CollaboRATE questionnaire. The qualitative part included a content analysis of notes from field studies of interactions between healthcare professionals and patients.

**Results:**

A total of 615 patients from an emergency department at a medium-sized hospital completed the questionnaire. Roughly, a third gave top-box scores (36%), indicating optimal involvement in decisions. Two factors, being discharged home and not readmitted, were significantly associated with the experience of being involved. In clinical practice, there was a focus on symptoms, and diagnostic tools and choice of treatment were decisive for the further care trajectory of the patients. Speed and low continuity left limited opportunities for dialogue to uncover patients' preferences. At the same time, the patients did not expect to be involved.

**Conclusions:**

Two out of three patients did not experience being involved in decisions regarding emergency department discharge. The interactions reflected an organizational structure in which the conditions for patient involvement were limited. Uncovering opportunities and initiatives to increase the number of patients who experience being involved in decisions is important tasks for the future.

## 1. Introduction

Many patients with various symptoms are admitted every day to emergency departments (EDs). Diagnostic activities are carried out, key decisions are made concerning the treatment, and care plans are decided within the first hours after arrival. After the initial evaluation patients will ultimately be discharged to home or admitted to a specialized ward. When planning discharge from the ED, there has been more than one reasonable solution for the individual patient [1]. At the same time, unmet care needs, such as insecurity about the treatment plan, lack of sufficient knowledge, and involvement of family, have been identified among both patients and relatives discharged from the ED [2].

Acutely admitted patients in seven European countries have reported that they felt that the healthcare professionals (HCPs) did not know what mattered most to them [3]. Studies conducted in the United States showed four out of five patients in the ED wanted to be involved in decisions [4], but less than half of the patients attending emergency care reported being involved to the degree they wanted [5]. In addition, patients in an American ED were waiting for an explicit invitation from HCPs to be involved in decisions [6]. Exploring elderly Danish patients' experiences at the emergency department showed HCPs being good at informing about further actions but too busy to ask the patients how they thought and felt about the situation [7]. At the same time, those readmitted within a week experienced the transition to home as unsafe and troublesome [8]. Furthermore, a Canadian study showed that, when patients were readmitted to the ED, patients' readiness and concerns were not integrated as part of the discharge planning according to the patients and liaison nurses [9].

Patient involvement in decisions is essential to deliver person-centered care (PCC), in which the patient is an active participant in decisions about their care plan. The goal of PCC is to include the patient´s preferences, needs, and values in clinical decisions [10, 11]. Through a meaningful dialogue where an exchange of information on options, benefits and harms, and what is important to the patient, the healthcare professional will involve the patient in decisions about planning care and treatment [10]. In other settings, having a PCC approach has been reported as being associated with improved health-reported outcomes such as fewer hospital visits, decreased length of hospital stays and higher quality-adjusted life-years [12], increased patients' feeling of security [13] and empowerment, improved functional outcomes, and enhanced satisfaction of care [14].

The extent to which patients in a Danish context experience involvement in discharge planning in acute care settings has not yet been explored, and in general, little is known about how patient involvement in decisions about discharge planning is managed in the clinical practice in ED.

Therefore, the present study aims to explore the extent of patients' involvement in discharge planning in acute care and to describe the context in which it takes place.

## 2. Materials and Methods

### 2.1. Design

The current study used a multimethod design [15], including both quantitative and qualitative data. A multimethod study combines different methods (quantitative and qualitative) and each study is planned and conducted to answer a particular subquestion [16]. Following this design, the two types of data were analyzed independently.

### 2.2. Setting and Population

The present study was conducted in an ED at a medium-sized hospital (867 beds) in the capital region of Denmark. On average, 233 patients are admitted to the ED per day, and three out of four patients are discharged from the ED directly to their home.

Before the patient arrives at the ED, they have been in contact with an HCP from the prehospital setting, who, based on a description of symptoms, defines an action diagnosis. This information is sent forward through an electronic system. A nurse is usually the first HCP the patient interacts with; the nurses assesses and prioritizes the urgency of treatment based on symptoms per the Danish Emergency Process Triage [17] and collects clinical data. During the trajectory of the patient, different HCPs are involved, and discharge planning can take place throughout the entire care trajectory in the ED.

#### 2.2.1. Quantitative Part

The participants were patients ≥18 years and consecutively included on randomly selected days just before leaving the ED. Patients were only included once and were excluded if their conditions were life threatening, they needed physical isolation, or they only had minor injuries. During the data collection, visitors were restricted because of the spreading infection of COVID-19 was in effect; so, only a few relatives were present.

#### 2.2.2. Qualitative Part

The participants were patients and HCPs in the ED. Patients with different health problems and different discharge plans in the ED were included, and the HCPs included were the ones caring for the patients in situations where the observations took place.

### 2.3. Data

#### 2.3.1. Quantitative Part

A translated and linguistically validated Danish version of the CollaboRATE [18] was used to measure the shared decision-making process in clinical practice. CollaboRATE contains three questions, each scored on a 10-point scale from zero to nine, with nine being the highest score [19]. Furthermore, the participants reported information about (a) whether they had held a dialogue about their discharge plan or not, (b) the profession of the HCP with whom they had held the dialogue, (c) their disposition plan (admitted or going home), and (d) living conditions. Additional data were collected from the participant's medical records and included gender, age, readmission within the last 30 days, diagnosis at discharge, Charlson comorbidity score (CCS) [20], and any registered psychiatric diagnosis. Data were given on an electronic tablet by the participants or by an HCP helping the participant. The data were entered directly into a Research Electronic Data Capture (REDCap) database.

#### 2.3.2. Qualitative Part

The data collection took place over a period of five months. The observer (first author) was in the ED for two to three hours at various times during the day for 12 randomly selected days. Field observations of when and how the planning of the discharge was carried out were conducted. Interactions between the patient and HCP were observed when the patient was arriving, during the stay, and just before leaving the ED. Field notes were written following an observational guide, including who was present and the content of their dialogues. Short informal individual interviews with the patients and HCPs, who were involved in the situations being observed, were carried out immediately after the observations to verify the observers understanding of the observations.

All data were collected between March 2021 and March 2022.

### 2.4. Data Analysis

#### 2.4.1. Quantitative Part

Statistical analysis was performed using the Statistical Package for Social Sciences version 25 (SPSS). Additional data were analyzed descriptively. The dependent variable, feeling involved or not feeling involved, was modeled by summarizing the scoring of all three questions in the CollaboRATE questionnaire and was thereafter dichotomized according to instructions [19]. Univariate and multivariate comparisons were performed using logistic regression. All response variables were first analyzed for univariate significance with the dependent variable (feeling involved), and variables that had a *p* value ≤0.1 were included in the multivariate model of analysis.

#### 2.4.2. Qualitative Part

The qualitative data were analyzed using content analysis with an inductive approach [21]. Field notes, including informal interviews, were coded, condensed, and then summarized into themes.

### 2.5. Ethical Considerations

The study was registered with the Danish Data Protection Agency (P-2020-1114). The Central Regional Committee on Health Research Ethics decided that no formal ethical approval was necessary (nr. 20068796). All participants gave informed consent after being informed both verbally and in writing. Data were handled anonymously and confidentially according to the Helsinki Declaration [22].

## 3. Results

### 3.1. Quantitative Part

**Figure 1:**
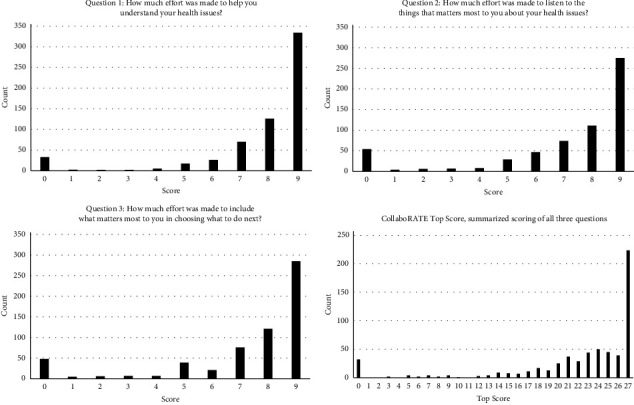
The distribution of the answers to the individual questions and the top score of CollaboRATE. The answers to the individual questions are ranged on a ten-point scale from 0 (no effort was made) to 9 (every effort was made). The top score is summarizing the score of all three questions, *n* = 615.

A total of 615 patients completed the CollaboRATE questionnaire. The characteristics of the participants in the quantitative part are found in Table 1. A vast number of participants (93%) indicated having held a dialogue in the ED with an HCP about the discharge plan. Out of these, 87% had a dialogue with a physician and the rest with a nurse. In total, 36% of the participants felt involved in planning the discharge. The distribution of the answers to the individual questions of CollaboRATE is presented in Figure 1.

Table 2 shows the outcomes of the univariate and multivariate logistic regression analyses. Two statistically significant predictors of feeling involved were found. The chances were 50% higher (CI 1.505–2.110) when it came to feeling involved in the discharge plan when the patient was going home than when the patient was being transferred to a specialized ward (*P*=0.018). Patients had a 92% higher chance of feeling involved (CI 1.251–2.942) if they had no previous admission within the past 30 days (*P*=0.003).

### 3.2. Qualitative Part

We conducted 26 hours of observations in the ED, with a focus on the interaction between the HCPs and patients. Furthermore, 14 patients and 11 HCPs were interviewed. In total, 46 typewritten pages with field notes and answers to questions constitute the material.

We found three themes describing the conditions for patient involvement in decisions regarding the discharge process in the ED: focus is on the symptoms, diagnostic tools and choice of treatment are decisive, and need for speed.

### 3.3. Focus Is on the Symptoms

In general, the attention in the dialogue between the patient and HCP was centered on the assessment of symptoms. Before arriving and during the entire care trajectory in the ED, the focus was on the patient's symptoms. Patients arriving in the ED were placed in a treatment room where the interactions with the HCPs took place and which was filled with different devices to track patients' symptoms.

The most common first question the patient was asked was related to symptoms and directly linked to detecting information about the physical state of the patient. Either the HCP would ask directly about symptoms, as illustrated by questions such as the following:

*Do you have stomachaches? (Field observation, nurse (15)); Have you experienced sensory disturbances? (Field observation, physician (28))*

Or the HCP would ask more openly, and the patient would describe symptoms, as illustrated by questions such as

*“Why are you here today?” (Field observation, nurse (25)),* followed by answers such as *“I am afraid of fainting” or “Having problems with my stomach” (Field observations)*.

The HCPs expressed how the cause of the patient's symptoms was not always connected to the physical symptoms, but detecting the genuine cause sometimes required a more in-depth dialogue with the patient to plan the right care.

*This patient's return to the ED is more about feeling alone because her husband has just moved to a nursing home, than about symptoms of constipation” (Informal interview, nurse (24))*

Although referring to missing time available in the clinical practice to have this dialogue with the patient, the nurses kept most of their focus on the medical symptoms. This was also observed in observations of clinical practice.

### 3.4. Diagnostic Tools and Choice of Treatment Are Decisive

The information on the patients' signs of illness was sent from the prehospital setting to the ED before the patient arrived, and this information initiated the interaction between the patient and HCP, even before they met. One nurse stated that she always used the information because it defined which tests and tasks she was expected to perform when interacting with the patient. In an observed situation, the nurse expressed what was needed according to the guidelines and what was *“… not a part of the package” (Field observation, nurse (25))*.

The patient's clinical signs of illness and results of the tests were decisive for the next step in the patient's trajectory. The HCPs described that, in their experience, patients did not expect to be involved in decisions regarding their discharge plan. Patients described that they respected “medical competence” and “medical expertise.” A patient directly said the following:

*“I shall not be involved” while describing that being admitted was fitting badly with his work and family life (Informal interview (8))*

The HCP explained how clinical guidelines, test results, and the clinical assessment of the patient were decisive in making the decision of whether a patient should be sent home or admitted to a specialized ward. In a situation where a patient was crying and did not want to go home, the physician said, *“This is an ED, and there is no space here” (Field observations (23)).* The physician subsequently prescribed a cardiogram, and the results of this test would decide if an admission could be justified.

This left limited conditions for dialogue between the HCP and patient related to the involvement of the patient's preferences, and few patients anticipated being involved.

### 3.5. Need for Speed

Speed characterized the work of the nurses. The nurses were usually either standing or in motion, except when they performed a task such as taking blood samples that required them to sit down.

There was low continuity, and seldom did the patient meet the same HCP more than once. The interaction between the nurse and patient was mainly focused on solving tasks such as giving medication, measuring blood pressure, or coordinating X-rays, and infrequently, it was observed that the nurses talked with the patient without a specific purpose related to the current illness. The aim was to quickly collect sufficient information that could be decisive for the patient's care trajectory with a minimum delay or give information about what the patient could expect next.

*One nurse said, that when she had completed the initial assessment, she only got back to that same patient if the patient called her on the alarm or if there were a defined bedside task to solve (Informal interview, nurse (24))*

Electronic screens in the staff office continuously showed with different colors and a timer where the patients were in the trajectory or said, in other words, “what are we waiting for” and “how many patients are expected to arrive.” These screens indicated what tasks were next and what had to be prioritized.

Interruptions of the interactions between the patient and HCP happened often, and the nurses explained how they experienced being obliged to disconnect the contact with the patient to answer phones and/or colleagues speaking to them face-to-face. The nurses were constantly alert to be called to another patient who needed acute care right away. A nurse explained, *“I do not have time for a conversation with the patient” (Informal interview, nurse (24, 2))* because there was an expectation of being interrupted and called to other more urgent tasks.

Simultaneously, patients were reluctant to interrupt HCPs.

*“Can I disturb the nurse to get some painkillers?” a patient discussed with his relative (Field observation (28)).*

The patient experienced how little time the nurse had for him when she was in motion delivering a cup of pills saying that she was off immediately again because an acutely ill patient needed her.

## 4. Discussion

In the present study, 36% of the patients felt involved in decisions regarding their discharge plan in the ED. CollaboRATE is not a time-consuming instrument for measuring the patient-perceived level of shared decision-making but rarely used in an ED context. A study from the United States found that 49% of patients felt involved in decisions in an ED setting and that the most common decision for which involvement in decisions was used was around ED disposition (admission vs. discharge) [5]. It is unknown if this difference in numbers is related to structural differences in healthcare delivery systems, but in both studies, the numbers are low. This might also be related to the acute and unforeseen situations the patients are in, making it more difficult to engage in care decisions.

Being discharged to their own homes and having no previous admission within 30 days were significant factors associated with patients feeling involved. The present study is the first to explore possible associations with feelings involved in the ED setting that we know of. Associations have been found in a primary care setting between feeling involved and increased age and being a female patient [23], which we did not find in this study. Knowledge about these factors might be useful in the work of increasing patients' experiences of involvement, but further investigation is needed to determine how the effort should be planned.

In our study, we found a clinical practice that was characterized by speed and low continuity, having a focus on the symptoms, diagnostic tools, and choice of treatment being decisive for whether the patient should be discharged or admitted. This left limited conditions for an exploration of the patient's subjective understanding of symptoms leading to hospitalization and inclusion of the patient's preferences in discharge planning. These findings are in line with a scoping review reporting how health system characteristics, such as care routines and workflow, influence the possibility of implementing involvement in decisions [24].

The results of this study and the findings of both the quantitative and the qualitative part in our study support each other, illustrating a clinical practice with a low focus on patient involvement in decisions and patients responding a low degree of being involved. Data thereby provide a multidimensional view of patient involvement in decisions when planning the discharge.

The healthcare system is evolving rapidly, and HCPs may find it difficult to keep up with knowledge on what care trajectories are available. In the Danish context, several new options regarding discharge have been implemented [25]; now, there are a broad variety of options to navigate: being admitted to a specialized ward; going home with a (subacute) outpatient consultation; going home with help from a specialist acute team; going home with help from primary healthcare; going home with a scheduled rehabilitation program or a rehabilitation stay; or going home with a recommendation to consult the family doctor. Furthermore, these options are differently organized in the individual municipality, making it even more complex.

In our study, we found that patients relied on HCPs to be experts and did not expect to be involved in decisions about their discharge plans. This differs from other studies abroad showing that patients in the ED want to be involved [4, 7], which might be linked to cultural differences and differences in the incentive structure of healthcare delivery systems. In addition, a PCC focus is important when decisions are made in the ED because, as expressed by the patients, an advantage for one person can be a disadvantage for another and vice versa [26]. When patients are not asking to be involved, this imposes a liability on the HCP to be the one inviting the patients to be involved in decisions. To support more systematic patient involvement in ED decisions, dialogue tools to initiate this objective might be useful.

Our study only focused on patient involvement in decisions regarding discharge planning, and involvement in other situations was not noted. Data were collected in one setting but might be transferrable across EDs in general. If the presence of COVID-19 and the various restrictions imposed in an on-off fashion during the data collection have influenced the trustworthiness of our results, it is not clear how. Perhaps, the greater work pressure, which occurred during the pandemic, has influenced the possibility of involving patients. To increase trustworthiness of findings, all authors participated in the analysis and interpretation of the data and continued the analysis until a consensus on final wording was reached.

Patients were excluded, who had cognitive impairment, who were not Danish speaking, and who were isolated due to the risk of infection. Together, these people represent a relatively high number of patients, and their perspectives are missing. In our study, there were a lower number of patients who were discharged to their homes (55.8%) than the overall group of patients admitted to the ED (72.8%). This is probably because of the exclusion the healthiest patients who were quickly out of the ED again. The CollaboRATE has shown good reliability [27], but further validation of the Danish version might be needed. Some patients were helped with completing the questionnaire by HCPs (including the first author). This may have influenced the results.

## 5. Conclusions

In the present study, two out of three patients did not feel being involved in decisions. The interaction between patients and HCPs reflected an organizational structure with a focus on symptoms, diagnostic tools, and choice of treatment being decisive, along with speed and low continuity, which limited the opportunities for patient involvement in decisions regarding their discharge planning. If patient involvement in decisions must become a current part of clinical practice, HCPs must take the initiative because the patient will not request to be involved. More research is needed on initiatives to uncover opportunities to facilitate a more systematic PCC approach and increase the number of patients feeling involved in decisions regarding discharge planning in the ED.

## Figures and Tables

**Table 1:** Characteristics of participants included in the quantitative part, *n* = 615.

Variables	
*Age mean (range)*	
Years	62.6 (18–96)
*Gender N (%)*	
Female	310 (50.4)
Male	305 (49.6)
*Discharge from the ED N (%)*	
Going home	343 (55.8)
Inpatient	272 (44.2)
*Living condition* ^*a*^ *N (%)*	
Living alone	240 (39.6)
Living with someone	366 (60.4)
*Diagnosis at departure* ^*b*^ *N (%)*	
Medical diagnosis	384 (62.7)
Surgical diagnosis	228 (37.3)
*Readmission within the last 30 days N (%)*	
No	478 (77.7)
Yes	137 (22.3)
*Psychiatric diagnosis N (%)*	
No	565 (91.1)
Yes	50 (8.1)
*Comorbidity (Charlson comorbidity index) N (%)*	
None	93 (15.1)
Comorbidity	522 (84.9)

^a^missing *n* = 9; ^b^missing *n* = 3.

**Table 2:** The univariate and multivariate odds ratio of patients feeling involved (*N* = 603).

Risk factors	*Univariate*		*Multivariate*	
	Odds ratio (95% CI)	*p*	Odds ratio (95% CI)	*p*
*Discharge from the ED*				
Going home vs. inpatient	1.481 (1.059–2.071)	0.022^*∗*^	1.505 (1.074–2.110)	0.018
*Gender*				
Female vs. male	0.961 (0.692–1.335)	0.813		
*Age (years)*				
≤60 vs. >60	0.916 (0.653–1.286)	0.613		
*Living condition*				
Living with someone vs. living alone	1.145 (0.815–1.608)	0.435		
*Diagnosis at departure*				
Surgical vs. medical	1.009 (0.718–1.419)	0.959		
*Readmission within the past 30 days*				
No vs. yes	1.889 (1.234–2.891)	0.003^*∗*^	1.919 (1.251–2.942)	0.003^*∗*^
*Psychiatric diagnosis*				
No vs. yes	1.359 (0.725–2.549)	0.338		
*Comorbidity (Charlson comorbidity index)*				
None vs. comorbidity	1.164 (0.730–1.855)	0.524		

^*∗*^Modeling the variables for the model of analysis reduced the sample to 612 because of missing data.

## Data Availability

The data used to support the findings of the study are available from the corresponding author upon request. The data are not publicly available because of privacy or ethical restrictions.

## Supplementary Materials

Supplementary Materials STROBE statement—checklist of items that should be included in reports of cross-sectional studies and COREQ (COnsolidated criteria for REporting Qualitative research) checklist.
